# Multiple value signals in dopaminergic midbrain and their role in avoidance contexts

**DOI:** 10.1016/j.neuroimage.2016.04.062

**Published:** 2016-07-15

**Authors:** Francesco Rigoli, Benjamin Chew, Peter Dayan, Raymond J. Dolan

**Affiliations:** a The Wellcome Trust Centre for Neuroimaging, UCL, 12 Queen Square, London WC1N 3BG, UK; b Gatsby Computational Neuroscience Unit, UCL, 17 Queen Square, London WC1N 3AR, UK; c Max Planck UCL Centre for Computational Psychiatry and Ageing Research, London WC1B 5EH, UK

## Abstract

The role of dopaminergic brain regions in avoidance behaviour is unclear. Active avoidance requires motivation, and the latter is linked to increased activity in dopaminergic regions. However, avoidance is also often tethered to the prospect of punishment, a state typically characterized by below baseline levels of dopaminergic function. Avoidance has been considered from the perspective of two-factor theories where the prospect of safety is considered to act as a surrogate for reward, leading to dopamine release and enhanced motivational drive. Using fMRI we investigated predictions from two-factor theory by separating the neural representation of a conventional net expected value, which is negative in the case of avoidance, from an adjusted expected value which factors in a possibility of punishment and is larger for both big rewards and big (predictably avoidable) punishments. We show that neural responses in ventral striatum and ventral tegmental area/substantial nigra (VTA/SN) covaried with net expected value. Activity in VTA/SN also covaried with an adjusted expected value, as did activity in anterior insula. Consistent with two-factor theory models, the findings indicate that VTA/SN and insula process an adjusted expected value during avoidance behaviour.

## Highlights

• We use fMRI and a novel visual-search paradigm to study the role of dopaminergic regions is avoidance contexts
• Activity in ventral striatum and ventral tegmental area/substantia nigra (VTA/SN) represents a raw expected value
• Activity in VTA/SN also reflects an expected value adjusted to the punishment potentially avoidable through action
• An adjusted expected value was also consistent with activity in insula

## 1 Introduction

Dopaminergic rich brain regions, including ventral striatum and midbrain ventral tegmental area/substantia nigra (VTA/SN), are implicated in evaluation and motivation (D'Ardenne et al., 2008, Kable and Glimcher, 2007, O'Doherty et al., 2004, Seymour et al., 2007, Tobler et al., 2005, Tom et al., 2007). In appetitive contexts, these regions show enhanced responses to larger rewards, consistent with representing expected value (EV) and reinforcement prediction error (RPE). Several findings link these regions with energizing effects or motoric vigour, in keeping with a link between EV and motivation (Berridge and Robinson, 1998, Niv et al., 2007, Salamone and Correa, 2002). However, evidence regarding dopaminergic involvement in avoidance contexts is scarce, and whether there is an increased activation in dopaminergic circuitry with punishment remains contentious (Boureau and Dayan, 2011, Dayan, 2012, Oleson and Cheer, 2013).

Evidence from Pavlovian conditioning paradigms (i.e., where acting is irrelevant; D'Ardenne et al., 2008) has shown that activity in dopaminergic regions is inhibited by larger punishments, suggesting inhibition in avoidance too (i.e., when acting is required to prevent punishment). On the other hand, avoidance studies report more vigorous motoric responses with larger punishments (Oleson and Cheer, 2013) and impaired avoidance behaviour with dopamine depletion (Cooper et al., 1973, Darvas et al., 2011, McCullough et al., 1993). Given an association between dopamine activity and motivation (Berridge and Robinson, 1998, Niv et al., 2007, Salamone and Correa, 2002), these findings hint at an increased dopaminergic response with larger punishment.

**Fig. 1:**
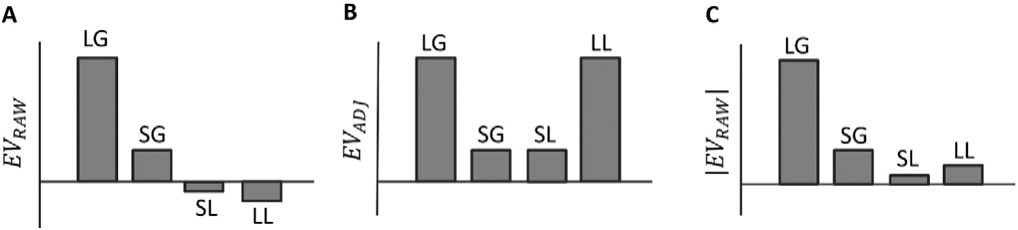
Representation of the raw EV (*EV*_*RAW*_; panel A), the EV adjusted to the level of punishment avoidable through action (*EV*_*ADJ*_; panel B), and the absolute value of the raw EV, corresponding to the absolute expected reinforcer (|* EV*_*RAW*_ |; panel C). Take a scenario where an agent has to perform an action in different conditions where (i) the action leads to a large monetary gain (LG; e.g., £10) with a given probability (e.g., 0.95, corresponding to the true participants' task performance) and not performing the action leads to zero outcome, (ii) performing the action leads to a small monetary gain (SG; e.g., £1) with a given probability (0.95) and not performing the action leads to zero outcome, (iii) performing the action leads to avoidance of a small monetary loss (SL; e.g. £1) with a given probability (0.95) and not performing the action leads to a small loss for sure, (iv) performing the action leads to avoidance of a large monetary loss (LL; e.g. £10) with a given probability (0.95) and not performing the action leads to a large loss for sure. *EV*_*RAW*_ corresponds to the value of the reinforcer multiplied by the probability of getting that reinforcer once the instrumental action is performed. This quantity decreases monotonically from LG, SG, SL to LL, as shown on panel A (e.g., LG: £10 × 0.95 = £9.5; SG: £1 × 0.95 = £0.95; SL: −£1 × 0.05 = −£0.05; LL: −£10 × 0.05 = −£0.5). *EV*_*ADJ*_ corresponds to the value of the reinforcer multiplied by the probability of getting that reinforcer minus the level of punishment (with negative sign) avoided by performing the instrumental action (multiplied by the probability of avoiding the punishment), and this is higher for LG and LL compared to SG and SL, as shown on panel B (e.g., LG: (£10 × 0.95)–£0 = £9.5; SG: (£1 × 0.5)–£0 = £0.95; SL = (−£1 × 0.05)–(−£1 × 0.95) = £0.9; LL = (−£10 × 0.05)–(−£10 × 0.95) = £9). From these graphs it can be seen that signalling *EV*_*RAW*_ or |* EV*_*RAW*_ | predicts a main effect of valence (i.e., (LG + SG)–(SL + LL), while signalling *EV*_*ADJ*_ predicts a main effect of amount (i.e., (LG + LL)–(SG + SL)). In addition, *EV*_*RAW*_ and |* EV*_*RAW*_ | predict an interaction effect in which the difference between a large and a small gain is larger than the difference between a large and a small loss (i.e., (LG − SG)–(LL − SL)). Therefore, here predictions associated with *EV*_*ADJ*_ can be dissociated from any influence exerted by *EV*_*RAW*_ or |* EV*_*RAW*_ |. This type of scenario is exploited in our experimental paradigm.

A recent computational model (Dayan, 2012), inspired by classical two-factor theory (Mowrer, 1947), suggests a potential resolution. In avoidance contexts, the model postulates an expected value signal which is adjusted to the level of punishment that is potentially avoidable through action. This adjusted signal is postulated as driving a dopaminergic response. In line with this, we designed a paradigm where we could distinguish between a raw EV signal (*EV*_*RAW*_) and an EV signal adjusted to the potentially avoidable punishment (*EV*_*ADJ*_). Crucially, these two value representations can be disentangled in the context of an instrumental task involving reward and punishment (Fig. 1). Consider a condition in which an animal has to perform an action to obtain a reward. Here, *EV*_*RAW*_ is equivalent to *EV*_*ADJ*_ since punishment is not involved. However, for performance of an action to avoid punishment, *EV*_*RAW*_ decreases with larger compared to smaller amounts of punishment (i.e., a less negative value is expected with smaller punishment) while *EV*_*ADJ*_ increases for larger compared to smaller punishment amounts because the level of avoidable punishment increases with larger punishment.

**Fig. 2:**
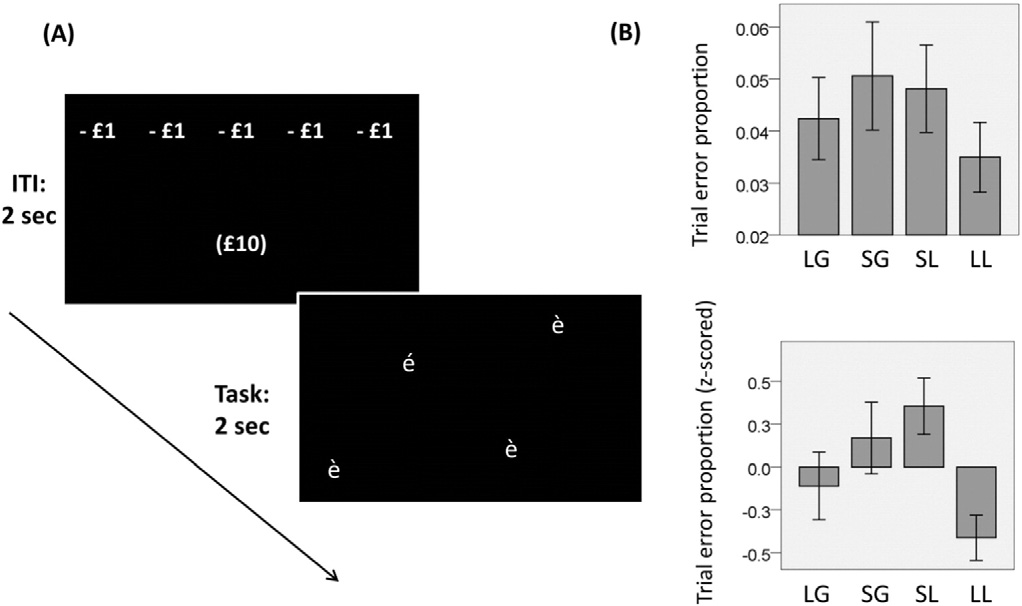
A: Experimental paradigm. Participants had to press a left/right button according to the location on the screen of a target presented among distractors. In different blocks, correct responses led to obtaining a large monetary gain (LG), a small monetary gain (SG), or avoiding a small monetary loss (LS) or a large monetary loss (LL). Before each trial, an information panel displayed (i) on the top of the screen, a row of monetary amounts corresponding to the condition of the current block *n* (the number of monetary amounts displayed corresponds to the number of trials remaining in the current blocks); and (ii) on the bottom of the screen, a monetary amount in brackets corresponding to the condition in the subsequent block *n* + *1*. Next, the target (*é*) and three distractors (*è*) were presented. Participants were required to press a left/right button corresponding to the side of the screen on which the target was displayed (left or right). B: Behavioural performance (corresponding to the proportion of uncorrect responses) as a function of the experimental conditions. Top: raw scores (with standard errors); bottom: Z-scores computed across the different conditions for each subject.

To investigate neural signatures of *EV*_*ADJ*_ independent of *EV*_*RAW*_, we considered instrumental behaviour in relation to gain or loss while manipulating their respective magnitudes. During functional magnetic resonance imaging (fMRI) recording, participants performed a task (Fig. 2A) requiring a right/left button press corresponding to the position on a screen of a visual target stimulus, presented together with distractors. On each trial, correct responses allowed participants either to gain or avoiding losing money associated with one of two amounts, as described by a two-by-two factorial design (factors of outcome valence (loss/gain) and monetary amount (£1/£10)). This design allowed us to test whether brain regions linked with an incentive value representation reflect *EV*_*RAW*_, *EV*_*ADJ*_, or both. A response in a region that represents *EV*_*RAW*_ would be expected to increase when contrasting reward with punishment (independent of amount), while a response in a region representing *EV*_*ADJ*_ would be expected to increase for large compared to small monetary amount (independent of valence).

It has been proposed that learning and performance of an avoidance response could usefully be differentiated, since the learning might involve two distinct signals, one associated with the possibility of the aversive outcome; the other with its rescindment (Dayan, 2012, Mackintosh, 1983, Mowrer, 1947). In order to focus on performance, we minimized any learning component by instructing participants on gain/loss contingencies and by allowing them to practise with the task before entering the scanner.

## 2 Methods

### 2.1 Participants

Twenty-two healthy right-handed human participants were recruited. Three subjects were excluded from analyses because of technical problems. Thus, the experimental sample included 19 subjects (9 females, mean age 27). All participants had normal or corrected-to-normal vision. None had history of head injury, a diagnosis of any neurological or psychiatric condition, or was currently on medication affecting the central nervous system. All participants provided written informed consent and were paid for participating. The study was approved by the University College of London Research Ethics Committee.

### 2.2 Experimental paradigm and procedure

Inside the MRI scanner, participants performed a computer-based task lasting approximately 35 min organized across two sessions of equal length (Fig. 2). On each trial, a target (corresponding to the letter *é*) and three distractors (corresponding to the letter *è*) appeared simultaneously on the screen, with the four stimuli shown in a randomized position with two of them appearing on each side of the screen. For each trial, participants had to press a right/left button on a keypad corresponding to the position of the target within 2 s. In some trials, correct responses were associated with a monetary gain of either £1 or £10 and incorrect responses were associated with no reward. In other trials, correct responses were associated with no monetary loss and incorrect responses with a monetary loss of either £1 or £10. Trials with equal valence (reward or punishment) and equal monetary amount (£1 and £10) were arranged in 32 blocks (each including 8 trials) ordered pseudo-randomly. During the intertrial interval, an information panel was presented for 2 s showing (i) the number of trials remaining in the current block *n*, represented as a row of equal outcome displayed on the top of the screen and (ii) the condition of the subsequent block *n* + *1* represented by an outcome displayed in the bottom of the screen in brackets. The latter component was included because we were initially interested in decorrelating in time the information about reward (linked with information about the subsequent block *n* + *1*) and the reward itself (linked with the reward condition of the current block *n*). However, no effect was found linked to the former component, and hence we do not discuss it further. After the information panel, the target and distractors were presented and remained on the screen for 2 s independently from reaction time, followed either by a new information panel or by an error feedback appearing for 1 s when participants pressed the wrong button or did not press at all.

Participants were tested at the Wellcome Trust Centre for Neuroimaging, at the University College London (UCL). At the beginning of the experiment, participants received an endowment of £17 and at the end of the experiment one outcome was randomly selected among those received and either added or subtracted from the endowment. Before scanning, subjects provided informed consent and were fully instructed on task contingencies and rules about the payment. Next they familiarized with the task outside the scanner for up to 100 unpaid trials.

Our task has similarities to an incentive delay paradigm (Knutson et al., 2001), but optimized in terms of participants' performance. Indeed, based in a pilot study, performance was expected to be at ceiling, entailing *EV*_*ADJ*_ was higher for large than small punishment and *EV*_*RAW*_ was higher for small than large punishment. This allowed us to isolate the two value signals. This optimized performance contrasts with the incentive delay task where performance is usually around 66% (Knutson et al., 2001), which means *EV*_*ADJ*_ and *EV*_*RAW*_ are roughly equivalent for large and small punishment and therefore harder to disentangle.

### 2.3 Imaging

The task was programmed with the Cogent toolbox (Wellcome Trust Centre for Neuroimaging) in Matlab. Blood oxygenation level dependent (BOLD) contrast functional images were acquired with echo-planar T2*-weighted imaging using a Siemens Trio 3-Tesla MR system with a 32 channel head coil. To maximize data in our regions of interest (ROIs), a partial volume of the ventral part of the brain was recorded. Image volumes consisted of 25 interleaved 3-mm-thick sagittal slices (inplane resolution = 3 × 3 mm; time to echo = 30 ms; repetition time = 1.75 s). The first six volumes acquired were discarded to allow for T1 equilibration effects. T1-weighted structural images were acquired at a 1 × 1 × 1 mm resolution. Data were analysed using Statistical Parametric Mapping (SPM) version 8 (Wellcome Trust Centre for Neuroimaging). Preprocessing included spatial realignment, unwarping using individual field maps, slice timing correction, normalization and smoothing. Specifically, functional volumes were realigned to the mean volume, were spatially normalized to the standard Montreal Neurological Institute (MNI) template with a 3 × 3 × 3 voxel size, and were smoothed with 8 mm Gaussian kernel. High-pass filtering with a cutoff of 128 s and AR(1)-model were applied.

Neural response was modelled using a canonical hemodynamic response function and a GLM including four boxcar function regressors associated respectively with large reward (+£10), small reward (+£1), large punishment (−£10) and small punishment (−£1). Four stick function regressors of no interest associated with the subsequent block condition were also included at block start (given that this information was provided in the panel), plus a stick function regressor indicating when an error response (i.e., a wrong or late button press) occurred. Note that regressors were uncorrelated due to the temporal gap between regressors associated with the current and subsequent block. Participants' respiration, heart rate and motion were included as nuisance regressors. Contrasts of interest were computed subject-by-subject and used for second-level one-sample t-tests across subjects. The reason we used t-tests is because, based on substantial literature (e.g., Bartra et al., 2013), we had precise hypotheses about the direction of effect to test in our ROIs. In other words, previous literature allowed us to test specifically for an increasing activation for positive minus negative valence, and for an increasing activation for large minus small monetary amounts.

We focus our analysis on ROIs within a priori dopaminergic rich regions, namely VTA/SN and ventral striatum, plus anterior insula, a region implicated in processing stimulus salience and motivation (Bartra et al., 2013, Critchley et al., 2001). Statistical testing followed small-volume correction (SVC) with a Family Wise Error (FWE) of p < 0.05. For VTA/SN, the ROI was manually defined using the software MRIcro and the mean structural image similar to the approach described in Guitart-Masip et al. (2011). Other ROIs were defined as spheres centred on coordinates extracted from a recent metanalysis of brain regions involved in representing EV and RPE (Bartra et al., 2013). For these spheres, a 8 mm diameter was used (e.g., De Martino et al., 2013), motivated by the a priori hypotheses about the location of the hypothesised effects within the ventral striatum and anterior insula.

## 3 Results

Participants' performance (Fig. 2B) was at ceiling (proportion of trial errors: mean = 0.044; SD = 0.032; range: 0.01–0.11). Considering the different conditions separately, the proportion of error trials was: for large gain, mean = 0.042, SD = 0.034; for small gain, mean = 0.051, SD = 0.045; for small loss, mean = 0.048, SD = 0.037; for large loss, mean = 0.035, SD = 0.029. Performance was not affected by outcome valence (F(1,18) = 0.913, p = 0.352) but was affected by outcome amount (F(1,18) = 7.005, p = 0.016), without evidence of an interaction effect (F(1,18) = 0.264, p = 0.614). Enhanced performance for large compared to small monetary amount can be interpreted as a motivational effect dependent on *EV*_*ADJ*_, while the absence of a difference in performance between reward and punishment conditions provides no support for a motivational effect of *EV*_*RAW*_.

We investigated a neural representation for different forms of value signal related to *EV*_*RAW*_, *EV*_*ADJ*_, or both. We used a simple general linear model (GLM) in which each condition was associated with a boxcar-function regressor with duration equal to block length, providing us with four regressors (large reward; small reward; large punishment; small punishment).

To dissociate encoding of *EV*_*RAW*_ and *EV*_*ADJ*_ (Fig. 1), we tested for a main effect of valence (consistent with *EV*_*RAW*_), a main effect of magnitude (consistent with *EV*_*ADJ*_), and an interaction between the two variables (consistent with *EV*_*RAW*_). Another possibility is that the expected magnitude of the reinforcer might be encoded in a manner corresponding to the absolute value of *EV*_*RAW*_ (|* EV*_*RAW*_ |; Fig. 1). This latter possibility makes predictions similar to the encoding of *EV*_*RAW*_, namely a main effect of valence and an interaction. Our design was not suited for dissociating *EV*_*RAW*_ and |* EV*_*RAW*_ | since it concentrated on testing for evidence that *EV*_*ADJ*_ was signalled, independent of both *EV*_*RAW*_ and |* EV*_*RAW*_ |.

**Fig. 3:**
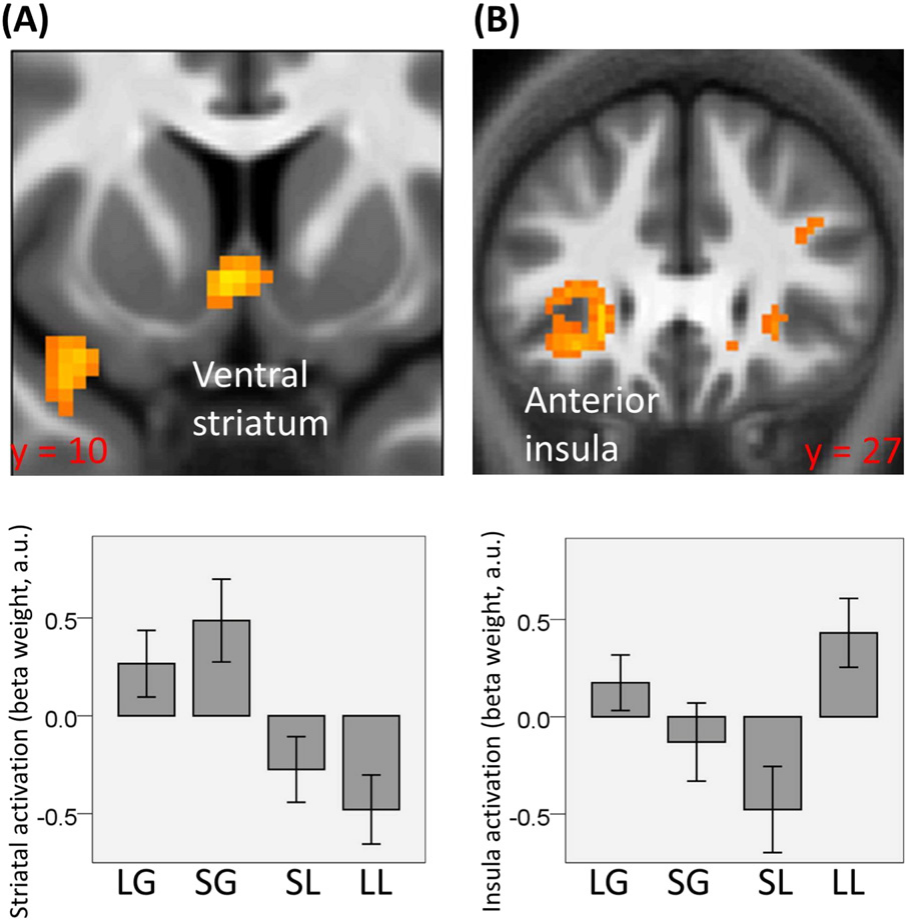
(A) Activity in ventral striatum for gains compared to losses (right: 2, 11, − 5; Z = 2.91, p = 0.049 SVC; left: − 3, 8, − 5; Z = 3.21, p = 0.024 SVC, in MNI space) independent of outcome amounts. On the bottom, beta weights of the different experimental conditions are reported for the peak-activation voxel in ventral striatum relative to the contrast gains minus losses. (B) Activity in left anterior insula for large compared to small outcome amounts (− 31, 18, − 10; Z = 3.38, p = 0.017 SVC) independent of outcome valence. On the bottom, beta weights of the different experimental conditions are reported for the peak-activation voxel in left anterior insula relative to the contrast large minus small outcome amounts.

**Fig. 4:**
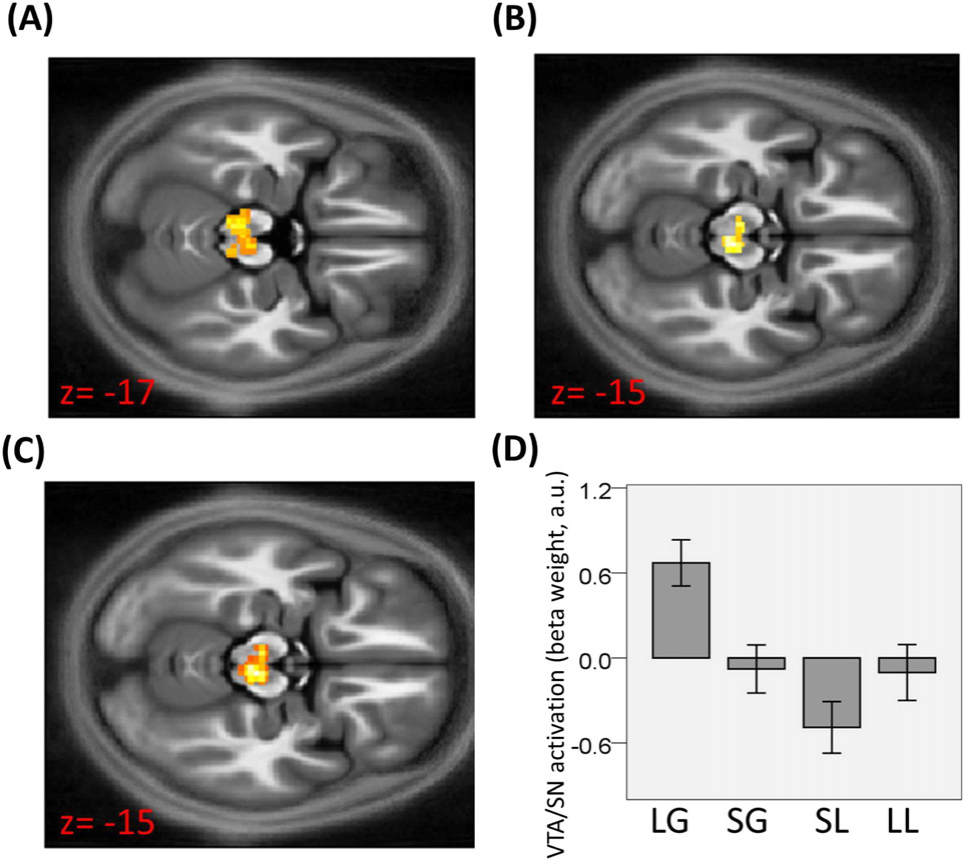
(A) Activity in VTA/SN for gains compared to losses (2, − 25, − 23; Z = 2.77, p = 0.040 SVC); (B) for large compared to small outcome amounts (5, − 27, − 15; Z = 3.08, p = 0.020 SVC); (C) for both contrasts according to a conjunction analysis (5, − 25, − 15; Z = 2.64, p = 0.043 SVC). (D) Beta weights of the different experimental conditions are reported for the peak-activation voxel in VTA/SN relative to the conjunction analysis.

When contrasting activity for rewards minus punishments (independent of large/small amounts), we observed an increase in bilateral ventral striatum (Fig. 3A; 2, 11, − 5; Z = 2.91, p = 0.049 SVC; left: − 3, 8, − 5; Z = 3.21, p = 0.024 SVC; in MNI coordinates space; see Methods for details on how ROIs were defined) and VTA/SN (Fig. 4A–D; 2, − 25, − 23; Z = 2.77, p = 0.040 SVC). When contrasting activity for large minus small outcome amounts (independent of the gain/loss condition), we observed increased activity in VTA/SN (Fig. 4B–D; 5, − 27, − 15; Z = 3.08, p = 0.020 SVC) but not ventral striatum. We also found a significant activation in left (but not right) anterior insula (Fig. 3B; − 31, 18, − 10; Z = 3.38, p = 0.017 SVC), a region important in motivation and in evaluating stimulus salience (Bartra et al., 2013, Critchley et al., 2001). Only at the most liberal threshold (p < 0.05 uncorrected) was activation for this contrast seen in left ventral striatum (− 17, 8, − 2; T = 2.64, p = 0.008 uncorrected) and right anterior insula (27, 28, − 3; T = 3.06, p = 0.004 uncorrected), though not in right ventral striatum, a result we report solely for completion.

There was no interaction in any of the above ROIs between factors of outcome valence and monetary amount, even at the most liberal p < 0.05 uncorrected threshold. In support of the conclusion that the lack of a valence–amount interaction effect is real and is not due to low statistical power, when comparing the difference between large and small gain against the difference between large and small loss, the voxel showing the highest t-value statistic within VTA/SN was associated with p > 0.4 uncorrected, and within anterior insula with p > 0.8 uncorrected.

We also investigated voxels showing a significant activation for both outcome valence and amount. These were tested with a conjunction analysis using a standard procedure (Nichols et al., 2005; in this method, an appropriate null hypothesis of a lack of a conjunction effect is implemented). This showed a significant effect in VTA/SN (Fig 4C–D; 5, − 25, − 15; Z = 2.64, p = 0.043 SVC).

We next examined whether the effect of outcome amount seen in the insula and VTA/SN was influenced by interactions among these regions. In a psychophysiological interaction (PPI) analysis with large compared to small outcome amount as modulating conditions and insula as seed region, we found a significant interaction effect in VTA/SN (− 3, − 25, − 20; Z = 3.22, p = 0.015 SVC as per above), that reflected an enhanced coupling between insula and VTA/SN with larger outcome amounts.

## 4 Discussion

In this study we separated the representation of two different expected value signals as posited with two-factor theory (Dayan, 2012, Mowrer, 1947). These comprise *EV*_*RAW*_, which is associated with pure outcome valence, and *EV*_*ADJ*_, an EV adjusted to encompass the amount of punishment that can potentially be avoided through action. We show neural responses in ventral striatum and anterior insula were consistent with representing *EV*_*RAW*_ and *EV*_*ADJ*_ respectively, while VTA/SN activation reflected both value signals.

Limitations implied by the use of fMRI mean that we cannot measure either neural activity or dopamine release and inferences that these effects are dopamine-mediated are necessarily indirect. Under an assumption that VTA/SN responses are reflective of dopaminergic activity, our findings support a role of this region in avoidance (Boureau and Dayan, 2011, Dayan, 2012, Oleson and Cheer, 2013). This is in keeping with data showing that animals fail to acquire, or perform, an avoidance response following lesions of either VTA/SN or ventral striatum, a deficit that is reversed following administration of dopaminergic drugs (Cooper et al., 1973, Darvas et al., 2011, McCullough et al., 1993, Zis et al., 1974). Along the same lines, microdialysis studies report enhanced dopamine levels in ventral striatum during learning and maintenance of avoidance behaviour (Dombrowski et al., 2013, McCullough et al., 1993). Furthermore, an enhanced response in ventral striatal neurons in receipt of dopaminergic projections is seen following presentation of warning signals associated with successful avoidance behaviour (Oleson et al., 2012).

Previous literature has not completely resolved whether increased engagement of dopaminergic regions is expected with larger punishments, when avoidance is possible (Boureau and Dayan, 2011, Dayan, 2012, Oleson and Cheer, 2013). This uncertainty arises out from the apparent conflicting idea that responses in these regions correlate with EV but also boost motivation. Here we clarify this issue, casting the problem in terms of a distinction between *EV*_*RAW*_ and *EV*_*ADJ*_ signals (Dayan, 2012). As predicted by a recent two-factor theory inspired model (Dayan, 2012), we show enhanced activity in dopaminergic VTA/SN for large, compared with small, punishments during an avoidance task, consistent with representing *EV*_*ADJ*_. One possibility is that such neural signal might be relevant for efficient performance of avoidance behaviour, as predicted by the model (Dayan, 2012). However, we stress that in our task we did not investigate for a direct relationship between this signal and actual performance of an avoidance response, and hence the prediction that brain representations of *EV*_*ADJ*_ affect behaviour remains to be empirically validated.

Participants were instructed about contingencies before the task in order to focus on the performance rather than the learning of the avoidance response (Mackintosh, 1983). Several theoretical and empirical considerations suggest that these two facets of avoidance may be dissociated, and indeed that learning is complicated by the involvement of at least two factors (Mackintosh, 1983, Mowrer, 1947). Our data leave open the question of how the value signals that emerged here in relation to performance are acquired during learning. Altogether, our results build on previous human and non-human studies that help clarifying the neural mechanisms underlying avoidance behaviour (Cooper et al., 1973, McCullough et al., 1993, Delgado et al., 2009, Dombrowski et al., 2013, Kim et al., 2006, Palminteri et al., 2012, Rigoli et al., 2012, Rigoli et al., 2016a, Rigoli et al., 2016b; Schlund and Cataldo, 2010, Schlund et al., 2011).

Salience can be conceived as the absolute reinforcer value (Bromberg-Martin et al., 2010), and a recent proposal has defined salience in terms of the absolute reinforcer value of all possible outcomes (multiplied by their probabilities; Esber and Haselgrove, 2011). This type of signal has been found to be relevant behaviourally and to be associated with activity in specific brain regions such as the temporo-parietal junction (Kahnt and Tobler, 2013). This conceptualization of salience has similarities with *EV*_*ADJ*_. However, a critical difference is a reliance on the concept of punishment potentially avoidable through action as considered in *EV*_*ADJ*_. This entails different predictions in our task. For instance, in the large loss condition, the model of Esber and Haselgrove (2011) would expect small salience given a small chance of a large loss due to ceiling performance, predicting the same pattern of activity as |* EV*_*RAW*_ |. This is not consistent with the observed response in VTA/SN which fits better with signalling an *EV*_*ADJ*_. One possibility is that different forms of salience are processed in the brain, with *EV*_*ADJ*_ representing a form of salience processed in dopaminergic midbrain. Notably, the fact that VTA/SN response might reflect *EV*_*ADJ*_ might explain why dopamine appears often to be related to behavioural relevance over and above its link with learning (Berridge and Robinson, 1998, Boureau and Dayan, 2011, Dayan, 2012, Niv et al., 2007, Oleson and Cheer, 2013, Salamone and Correa, 2002).

The VTA/SN showed greater activity for reward compared to punishment, an effect that appears to be unrelated to overt behaviour in our task, since there was no difference in performance with reward and punishment conditions. One possibility is that different value signals associated with specific reference points overlap in VTA/SN as this region would represent both *EV*_*ADJ*_ and *EV*_*RAW*_, resulting in a greater response for the large reward amount condition compared to the large punishment amount condition. Whether the interpolation of the two signals happens in single units or is segregated across the region remains a question for future research, though our observation of voxels recruited by both value signals hints at least a partial overlap.

There is debate as to whether VTA/SN activity reflects the expected absolute reinforce magnitude, corresponding to |* EV*_*RAW*_ |. In the context of our task, |* EV*_*RAW*_ | can be dissociated from *EV*_*ADJ*_ but not from *EV*_*RAW*_. Therefore, while our design allows us to reveal independent neural signatures of *EV*_*ADJ*_, it is not suitable to distinguish whether *EV*_*RAW*_ or |* EV*_*RAW*_ | is signalled in VTA/SN. Encoding |* EV*_*RAW*_ | requires a response correlated with outcome amount in instrumental paradigms (as used here) but also in Pavlovian paradigms, i.e., when acting has no consequences. In line with an influence of |* EV*_*RAW*_ |, single-cell recording studies have reported increased firing rates with both unexpected punishments and rewards in a sub-set of VTA/SN neurons during Pavlovian conditioning (Bromberg-Martin et al., 2010, Matsumoto and Hikosaka, 2009). However, fMRI data (representing a more global population response compared to single-cell studies) on this question are mixed. Studies using monetary incentives show decreased VTA/SN activity for large compared to small (unexpected) punishment in the context of a Pavlovian paradigm (D'Ardenne et al., 2008, Seymour et al., 2007), while studies using painful stimuli report increased activity in dopaminergic regions for larger painful stimuli (Seymour et al., 2007).

Our findings indicate that ventral striatal activity correlated with the outcome valence associated with *EV*_*RAW*_. However, since avoidance was overwhelmingly successful (with an average performance of 95%), the numerical value of *EV*_*RAW*_ would actually have been close to zero for losses. This could suggest that the representation of *EV*_*RAW*_ was non-linear, although there was no direct evidence of this in the observed striatal responses. Note that, as for VTAS/SN, our design is not suitable to clarify whether the response in striatum depended on *EV*_*RAW*_ or |* EV*_*RAW*_ |. We also failed to detect any significant effect linked with outcome amount connected with *EV*_*ADJ*_, in the ventral striatum. However, these data should be treated with caution because an effect in ventral striatum of outcome amount emerged using the most liberal significance threshold, raising the possibility that our null finding might be explained by lack of power. It should also be noted that a previous study has reported increased ventral striatal responses following presentation of warning signals associated with successful avoidance behaviour (Oleson et al., 2012). Moreover, it has been shown that response in ventral striatum depends more on the instrumental action required (i.e., go vs no-go) than on valence (Guitart-Masip et al., 2011).

It is well-established that activity in anterior insula is influenced by EV (Bartra et al., 2013, Critchley et al., 2001). Our findings fit with previous evidence indicating a response in this region both for large compared to small reward, and for large compared to small punishment. This has been interpreted as anterior insula encoding |* EV*_*RAW*_ |, namely the expected absolute reinforcement (Bartra et al., 2013). However, our data suggest that response in insula might reflect also *EV*_*ADJ*_. This finding is also linked with the idea that insula activity is linked to behavioural salience, as *EV*_*ADJ*_ incorporates action-relevant information in terms of punishment potentially avoidable (Critchley et al., 2001). Note also that we observed a change in coupling between insula and VTA/SN as a function of outcome amount. One plausible hypothesis is that insula integrates information about task salience, and through projections to dopaminergic neurons in VTA/SN, regulates their excitability in response to representations of *EV*_*ADJ*_.

In sum, we investigated value signalling in the context of avoidance behaviour and highlight a central role of VTA/SN and anterior insula in representing an EV representation adjusted to the level of punishment potentially avoidable through action.
